# Modeling Inherited Cardiomyopathies in Adult Zebrafish for Precision Medicine

**DOI:** 10.3389/fphys.2020.599244

**Published:** 2020-11-19

**Authors:** Yonghe Ding, Haisong Bu, Xiaolei Xu

**Affiliations:** ^1^Department of Biochemistry and Molecular Biology, Mayo Clinic, Rochester, MN, United States; ^2^Department of Cardiovascular Medicine, Mayo Clinic, Rochester, MN, United States; ^3^Clinical Center for Gene Diagnosis and Therapy, The Second Xiangya Hospital of Central South University, Changsha, China

**Keywords:** cardiomyopathy, adult zebrafish, causative gene, animal model, precision medicine

## Abstract

Cardiomyopathies are a highly heterogeneous group of heart muscle disorders. More than 100 causative genes have been linked to various cardiomyopathies, which explain about half of familial cardiomyopathy cases. More than a dozen candidate therapeutic signaling pathways have been identified; however, precision medicine is not being used to treat the various types of cardiomyopathy because knowledge is lacking for how to tailor treatment plans for different genetic causes. Adult zebrafish (*Danio rerio*) have a higher throughout than rodents and are an emerging vertebrate model for studying cardiomyopathy. Herein, we review progress in the past decade that has proven the feasibility of this simple vertebrate for modeling inherited cardiomyopathies of distinct etiology, identifying effective therapeutic strategies for a particular type of cardiomyopathy, and discovering new cardiomyopathy genes or new therapeutic strategies via a forward genetic approach. On the basis of this progress, we discuss future research that would benefit from integrating this emerging model, including discovery of remaining causative genes and development of genotype-based therapies. Studies using this efficient vertebrate model are anticipated to significantly accelerate the implementation of precision medicine for inherited cardiomyopathies.

## Introduction

Cardiomyopathy refers to a group of heterogeneous heart muscle disorders that cause cardiac dysfunction. Cardiomyopathy is the most common cause of heart failure and afflicts millions of people worldwide (Lipshultz et al., 2019). Non-ischemic cardiomyopathy can be broadly classified, based on clinical features, into hypertrophic cardiomyopathy (HCM), dilated cardiomyopathy (DCM), restrictive cardiomyopathy (RCM), and arrhythmogenic cardiomyopathy (ACM) (Maron et al., 2006). HCM, the most common form of genetic heart disease, is characterized by left ventricular (LV) hypertrophy (thickening) with diastolic dysfunction (estimated incidence, 1 in 500 persons) (Makavos et al., 2019). Major diagnostic criteria for HCM is a wall thickness ≥15 mm in one or more LV myocardial segments. Characteristic changes on electrocardiography may include repolarization changes, T-wave inversions, and abnormal Q waves (Gersh et al., 2011; Authors/Task Force members, Elliott et al., 2014). DCM, the most common type of non-ischemic cardiomyopathy, is characterized by an enlarged ventricular chamber, thinned ventricular walls, and systolic dysfunction (estimated prevalence, 1 in 250 persons) (Rosenbaum et al., 2020). Clinical diagnosis of DCM is based on two major criteria: (1) LV fractional shortening is <25% and/or LV ejection fraction is <45%; and (2) LV end-diastolic diameter is >117% of predicated value corrected for age and body surface area (Bozkurt et al., 2016; Pinto et al., 2016). RCM, the rarest form of cardiomyopathy, is characterized by diastolic dysfunction but normal or near normal systolic function and restrictive ventricular filling pattern on echocardiography (estimated prevalence, 1 in 5,000 persons) (Muchtar et al., 2017). ACM, another rare cardiac muscle disorder, is characterized by structural abnormalities occurring mainly in the right ventricle and ventricular arrhythmia (estimated prevalence, between 1 in 1,000 and 1 in 5,000 persons) (Mattesi et al., 2020). Major diagnostic characteristics of ACM include global or regional dysfunction and structural alterations, fibrous replacement of the right ventricle (RV)-free wall myocardium with or without fatty replacement of tissue on endomyocardial biopsy, repolarization abnormalities, depolarization/conduction abnormalities, arrhythmias, and family history (Marcus et al., 2010; Towbin et al., 2019).

Distinct phenotypes of cardiomyopathies could be partially explained by genetic heterogeneity. Since the discovery of *MYH7*, the first causative gene for HCM, more than 100 genes have been linked to cardiomyopathies (Geisterfer-Lowrance et al., 1990; Tanigawa et al., 1990; Marian and Braunwald, 2017; Muchtar et al., 2017; Yotti et al., 2019; James et al., 2020). Beyond a monogenic disease, findings from next-generation sequencing (NGS) suggest multiple genetic hits could contribute to phenotypic severity of cardiomyopathies (Xu et al., 2010; Harakalova et al., 2015; Kolokotronis et al., 2019). Both phenotypic and genetic heterogeneity strongly suggest that precision medicine should be practiced to treat cardiomyopathies of various causes (Fatkin et al., 2019). However, standard treatments are still the norm because insufficient knowledge exists to individualize treatment with precision medicine.

Animal models of cardiomyopathy are needed for comparing phenotypes of cardiomyopathies of various causes, deciphering the mechanisms of disease, and developing effective therapeutic strategies. Rodents are classic vertebrate models that have contributed substantially to our knowledge of cardiomyopathies. More than a dozen potential therapeutic pathways and related compounds have been identified, some of which have already been translated clinically (Ashrafian et al., 2011; Van Berlo et al., 2013; Kieserman et al., 2019; Pinilla-Vera et al., 2019; Repetti et al., 2019). However, the high cost and low throughput associated with rodent models has impeded their use in developing precision medicine. For example, it would be a daunting task to assess all known candidate therapies systematically in each rodent cardiomyopathy model of a particular genetic cause. Therefore, a reliable vertebrate model with higher throughput was needed.

Zebrafish are small tropical fish that are highly similar genetically to humans (Howe et al., 2013). Many unique advantages of zebrafish embryos, including transparency, make the zebrafish a useful and important model for genetic studies. Early studies using this model focused mainly on cardiac development and congenital heart disease (Bournele and Beis, 2016). The first reports describing embryonic zebrafish as cardiomyopathy models were published in 2002, when positional cloning of two mutations, *pickwick* and *silent heart*, identified *titin* and *tnnt2*, both known causative genes for cardiomyopathy (Sehnert et al., 2002; Xu et al., 2002). Additional studies using zebrafish embryos helped in the discovery of other cardiomyopathy genes and new therapeutic strategies (Bakkers, 2011; Gut et al., 2017). However, the studies were limited because embryo studies cannot mimic the age dependent penetrance of cardiomyopathies that often end in overt heart failure in adulthood. In 2009, cardiac remodeling phenotypes were first reported in an anemia mutant zebrafish, owing to a *band3* mutation, which underscored the feasibility of adult zebrafish as a simple vertebrate model for cardiomyopathy (Sun et al., 2009). Additional models have been generated for acquired cardiomyopathies, including those caused by doxorubicin, a common chemotherapy drug that can induce dose-dependent cardiotoxicity (Ding et al., 2011), and by phenylhydrazine hydrochloride, which can cause acute hemolysis and chronic anemic stress on the heart (Sun et al., 2009; Jopling et al., 2012). The advent of transgenic technology and genome editing technology further led to the generation of a panel of adult zebrafish models for inherited cardiomyopathies, which are the focus of the present review (Table 1).

**TABLE 1:** Adult zebrafish models of inherited cardiomyopathies.

Gene	Human phenotype	Mutation	Model type	Zebrafish phenotypes	References
*MYL3*	HCM	Premature stop at Y186	ENU/KO	Increased heart rate and systolic dysfunction	Scheid et al., 2016
*MYH6*	HCM	Frameshift at D1341, H1524	ENU/KO	Chamber dilation and weak atrial beat	Sarantis et al., 2019
*LAMP2*	HCM	Frameshift at A32	TALEN/KO	Chamber dilation, increased trabecular muscle density, diastolic dysfunction, hypercontractility, and increased mortality	Dvornikov et al., 2019
*DNAJB6*	DCM	Tol2 insertion	GBT/KO	Chamber dilation and cardiomyocyte hypertrophy	Ding et al., 2013, 2016
*GATAD1*	DCM	Frameshift at R67, T75	TALEN/KO	Reduced survival and reduced swimming capacity	Yang et al., 2016
		S102P	OE/Tg	Enlarged ventricle and outflow tract	
*BAG3*	DCM	Frameshift at P108	TALEN/KO	Chamber dilation, decreased trabecular muscle density, systolic dysfunction, hypocontractility, and increased mortality	Ding et al., 2019
*TTN*	DCM, HCM	Frameshift at K26331	TALEN/KO	Chamber dilation, QRS prolongation, impaired contractile response, reduced diastolic filling with hemodynamic stress, and increased mortality	Huttner et al., 2018
*SCN5A*	DCM, SSS	D1275N	OE/Tg	Bradycardia, conduction-system abnormalities, episodes of sinus arrest, and premature death	Huttner et al., 2013; Yan et al., 2020
*JUP*	ACM	2057del2	OE/Tg	Cardiomegaly, thinning of atrial and ventricular walls, peripheral edema, and increased mortality	Asimaki et al., 2014a
*ILK*	ACM	H77Y, P70L	OE/Tg	Reduced survival, fraction shortening and action potential, and accumulation of epicardial fat tissue	Brodehl et al., 2019
*KCNJ8*	Cantú syndrome	V65M	CRISPR-Cas9/KI	Enlarged ventricular chamber volume	Tessadori et al., 2018
*PITX2*	Atrial fibrillation	Frameshift at H49	TALEN/KO	Reduced cardiac function, arrhythmia, atrial conduction defects, sarcomere disassembly, and altered cardiac metabolism	Collins et al., 2019

*ACM, arrhythmogenic cardiomyopathy; CRISPR/Cas9, clustered regularly interspaced short palindromic repeats (CRISPR) and CRISPR-associated protein 9; DCM, dilated cardiomyopathy; ENU, N-ethyl-N-nitrosourea; GBT, gene-break transposon; HCM, hypertrophic cardiomyopathy; KI, knock-in; KO, knock-out; OE, overexpression; SSS, sick sinus syndrome; TALEN, transcription activator-like effector nucleases; Tg, transgenic.*

Different from lower model organisms such as *Drosophila*, a zebrafish heart has conserved cardiomyocytes with those in humans, as well as intact endocardium and epicardium. Electrophysiology of a zebrafish heart has higher conservation than mouse with humans (Bakkers, 2011; Macrae and Peterson, 2015; Gut et al., 2017). Heart rate is around 100 beats per minute (bpm), which resembles to human better than the mouse. Because a zebrafish heart is only sized about 1–2 mm in diameter, novel phenotyping toolkits, such as high-frequency echocardiography, the Langendorff *ex vivo* system, ECG, and single-myofibril contractility analysis, have been developed to define progression of cardiac remodeling in zebrafish (Dvornikov et al., 2014; Lin et al., 2015; Koth et al., 2017; Merrifield et al., 2017; Wang et al., 2017; Stoyek et al., 2018; Zhang et al., 2018; Wakamatsu et al., 2019; Yan et al., 2020). Because these toolkits have already been comprehensively reviewed recently (Dvornikov et al., 2018; Lin et al., 2020), we only list some new techniques in Table 2. Partially because these new technologies are not readily accessible, many published adult zebrafish inherited cardiomyopathy models have not been sufficiently phenotyped to be categorized into a certain type of cardiomyopathy. Therefore, we decided to group these models based on the type of cardiomyopathy that is associated with its human ortholog, such as HCM, DCM, ACM, and cardiomyopathy genes associated with complex syndromes. We did not include the RCM group because no adult zebrafish model for a human RCM gene has been reported yet.

**TABLE 2:** Emerging phenotyping techniques for adult zebrafish hearts.

Techniques	Major measurement parameters	Advantage	Disadvantage	References
HFE	Cardiac structure, cardiac function, stroke volume/output, blood flow, and hemodynamic	Non-invasive, close to physiological condition, and *in vivo*	Require anesthesia, heart rate interference, reliability issue, and quantification error variation	Wang et al., 2017
MRI	Heart anatomy and cardiac function	Non-invasive, close to physiological condition, *in vivo*, and high resolution	Require anesthesia, heart rate interference, reliability issue, and quantification error variation	Koth et al., 2017; Merrifield et al., 2017
*Ex vivo*	Cardiac pump function and stoke volume/cardiac output	Can reveal intrinsic pump potential, allow the control of flow, and suitable for acute study	Need to Isolate the heart and non-physiological condition	Zhang et al., 2018
ECG	Heart rate, QT-interval, p-wave, QRS complex, and T-wave	Simple, non-invasive, and quick assay	Require anesthesia	Lin et al., 2018; Yan et al., 2020
Swimming tunnel	Exercise capacity	Simple and non-invasive	Indirect implication of cardiac output/function	Wakamatsu et al., 2019
Voltage and calcium mapping	Action potential durations, atrial-ventricle delay, and calcium transients	Voltage and calcium dynamics	*In vitro* condition	Lin et al., 2015; Stoyek et al., 2018

*ECG, electrocardiogram; HFE, high-frequency echocardiography; MRI, magnetic resonance imaging.*

## Known Inherited Cardiomyopathy Genes

### HCM Genes

Because most causative genes for HCM encode sarcomeric proteins, HCM is also considered a disease of the sarcomere (Seidman and Seidman, 2011). Pathogenic sequence variants in *MYH7* and myosin-binding protein C (*MYBPC3*) account for approximately 75% of all inherited cases of HCM (Burke et al., 2016). Patients harboring sequence variants in genes that lead to lysosomal storage disease, such as lysosomal-associated membrane protein 2 (*LAMP2*), also manifest HCM phenotypes (Fu et al., 2016). To date, adult zebrafish models for the following three HCM genes have been reported.

#### MYL3

Essential myosin light chain 3 (*MYL3*), encoding a ventricular/cardiac isoform myosin essential light chain, has been identified as a causative gene of familial HCM, accounting for up to 5% of HCM cases (Olson et al., 2002; Keren et al., 2008). Scheid et al. (2016) reported phenotypic characterization of a heterozygous *lazy susan* (*laz^*m*647^*) mutant, which harbors a nonsense mutation in *myl3*. Like *pickwick* and *silent heart, laz^*m*647^* was originally identified as an embryonic lethal mutant from a large-scale mutagenesis screen (Stainier et al., 1996). Reduced cardiac contraction was noted in homozygous *laz*^–/–^mutants (Meder et al., 2009). By using non-invasive echocardiography and swimming exercise assays, Scheid et al. (2016) reported systolic dysfunction in adult heterozygous *laz* (*laz*^±^) zebrafish at a basal level, which became more severe upon physical stress, as reflected by increased sudden death. S195 phosphorylation of essential light chain is required for adaptation of cardiac function to augmented physical stress. Thus, heterozygous *laz* was the first inherited cardiomyopathy model reported in adult zebrafish. However, more detailed phenotyping of this model is needed to determine whether *laz* is a HCM model.

#### MYH6

In contrast to the *MYH7* gene that encodes the ventricular/slow myosin heavy chain β isoform, *MYH6* encodes the myosin heavy chain α isoform that is predominantly expressed in human cardiac atrium. Mutations in *MYH6*, although relatively rare, have been associated with both HCM and DCM (Schiaffino and Reggiani, 1996; Parker and Peckham, 2020). In a zebrafish heart, there are three major *MYH* homologs: atrial myosin heavy chain (*amhc*, also termed *myh6*), ventricular myosin heavy chain (*vmhc*), and ventricular myosin heavy chain-like (*vmhcl*) (Auman et al., 2007). While *amhc* specifically expresses in the atrium, the latter two genes express mostly in the ventricle (Shih et al., 2015). Sarantis et al. (2019) reported the characterization of the adult cardiac phenotype of *weak atrium* mutants that harbor frameshift mutations in *amhc*. They reported that atria remained hypoplastic and showed elastin deposition, whereas ventricles exhibited increased chamber size due to hyperplasia. Similar to *laz*, more detailed phenotyping of this model is needed to determine whether *weak atrium* exhibits HCM phenotypes.

#### LAMP2

Lysosome-associated membrane protein 2 (*LAMP2*) encodes a type I integral membrane protein that is localized to lysosomes and late endosomes. *LAMP2* was initially identified as a causative gene for Danon disease, manifesting with peripheral skeletal myopathy, intellectual disability, hepatic involvement, and retinopathy. Later, mutations in *LAMP2* were found in about 1% of patients with HCM (Danon et al., 1981; Maron et al., 2009). Using adult zebrafish as a vertebrate model, Dvornikov et al. (2019) introduced a truncational mutation into exon 2 of the *lamp2* gene by using transcription activator-like effector nuclease (TALEN)–based genome editing technology. In a *lamp2^*e*2/e2^* homozygous mutant at 10 months of age, there was increased density of the trabecular muscle, a thickened compact layer, fibrosis, and reduced ejection fraction at low flow that was ascribed to decreased end diastolic volume/body weight ratio. Also described were increased maximal isometric tension and accelerated actomyosin activation kinetics at the single-myofibril level, suggesting myofibrillar “hypercontractility.” Because many of these phenotypes are characteristic features of HCM in human patients and mammalian models, this study provided a starting point to define HCM-like phenotypic traits in adult zebrafish.

### DCM Genes

To date, more than 50 genes have been linked to human DCM. They can be broadly categorized into genes encoding sarcomere, cytoskeletal, mitochondrial, desmosomal, nuclear membrane, and RNA-binding proteins (Yotti et al., 2019). The three DCM genes described below have been modeled in adult zebrafish.

#### TTN

Truncation mutations in the *TTN* truncating variants (*TTNtv*) are the most common causative genetic lesion for inherited DCM, presenting in 15 to 20% of cases (Herman et al., 2012; Schafer et al., 2017). Allelic heterogeneity has been noted in *TTNtv* DCM. Whereas *TTNtv* in the C-terminal A-band region are pathogenic, *TTNtv* in the N-terminal Z-disk region are likely benign. In zebrafish, 2 *TTN* homologs are located in tandem on chromosome 14, termed *ttn.1* and *ttn.2* (Seeley et al., 2007). Huttner et al. (2018) generated zebrafish mutants harboring A-band *ttn.2* truncations that mimicked mutations found in two unrelated human probands with familial DCM. Whereas the homozygous *ttn.2* mutants exhibited severe cardiac dysmorphogenesis and premature death, heterozygous mutants (*ttn.2^*tv/*+^*) survived into adulthood and spontaneously developed DCM-like phenotypes, including reduced baseline ventricular systolic function, prolonged isovolumic relaxation, and increased diastolic passive stiffness in the absence of myocardial fibrosis. Thus, both systolic and diastolic dysfunction were noted in this zebrafish model of *TTNtv* cardiomyopathy.

#### BAG3

The BCL2-associated athanogene 3 (*BAG3*) gene encodes a co-chaperone protein that regulates unfolded protein aggregation and autophagy (Meriin et al., 2018). Mutations in the *BAG3* gene account for 2 to 7% of DCM cases, representing a common DCM causative gene (Norton et al., 2011; Franaszczyk et al., 2014; Dominguez et al., 2018). We recently generated zebrafish *bag3* gene knockout mutants by introducing frameshift mutations through TALEN-based genome editing technology (Ding et al., 2019). We characterized phenotypic traits of this model thoroughly by combining emerging technologies of high-frequency echocardiography, *ex vivo* heart pump function assays, and biophysical assays at the single-myofibril level. In *bag3^*e*2/e2^* homozygous mutants at 6 months of age, we detected ventricular chamber enlargement, reduced trabecular muscle density, and reduced ejection fraction ascribed to increased end-systolic volume/body weight that resembled the eccentric hypertrophy characteristic of DCM in mammals (Merlo et al., 2018). We also detected decreased maximal isometric tension and reduced activation of myofibril kinetics at the single-myofibril level, suggesting “hypocontractility.” Because many phenotypic traits are characteristic features of DCM in human patients and mammalian models, this study provides a starting point to define DCM-like phenotypic traits in adult zebrafish models.

#### GATAD1

The GATA zinc finger domain containing 1 (*GATAD1*) gene encodes a transcription factor that is more highly expressed in women than men with DCM (Heidecker et al., 2010). A homozygous recessive missense mutation, S102P, was discovered by locus mapping and whole exome sequencing in a family with adult-onset DCM (Theis et al., 2011). Yang et al. (2016) generated a *gatad1* knockout zebrafish mutant by TALEN technology as well as a transgenic fish line overexpressing the human *GATAD1*-S102P variant in cardiomyocytes. While *gatad1* knockout fish exhibited a mild heart failure–like phenotype after stress, the cardiomyocyte-specific overexpressing *GATAD1*-S102P transgenic fish have increased mortality, concurrent with a substantially enlarged ventricular chamber and decreased papillary muscle density, suggesting cardiac remodeling.

Cardiac phenotypes for many existing cardiomyopathy models have not been characterized in detail, partially because phenotyping tools are not readily available. Two of the most comprehensively characterized cardiomyopathy models in adult zebrafish are *lamp2^*e*2/e2^* and *bag3^*e*2/e2^* mutants. Common cardiomyopathy traits have been identified with this model, such as reduced ejection fraction, reduced exercise capacity, and aberrant expression of molecular markers, as have unique phenotypic traits that can discern DCM from HCM-like phenotypes. First, trabecular muscle density is reduced in the *bag3* knockout zebrafish, whereas it is increased in the *lamp2* knockout model. Similarly, Abdul-Wajid et al. (2018) recently used the percentage of ventricle covered in trabeculation as an index to determine HCM phenotype in the *jag2b* mutant adult zebrafish. Whether trabecular muscle density can indeed serve as a surrogate index of ventricular wall thickness in mammals, a key phenotypic trait that discerns DCM from HCM, remains to be established. Second, the *lamp2* knockout model, but not the *bag3* knockout model, had a rounded heart shape. Third, at the single-myofibril level, myofibrils from the *bag3* knockout model manifested hypocontractility, but myofibrils from the *lamp2* knockout model manifested hypercontractility. This phenomenon is consistent with that seen via biophysical analysis in mammalian DCM and HCM models (Davis et al., 2016; Spudich et al., 2016). In the Table 3, we summarized major phenotypic traits of human cardiomyopathies and compared with features and surrogate indices in corresponding adult zebrafish models. More inherited cardiomyopathy models need to be developed and comprehensively phenotyped, which will better define phenotypic traits among cardiomyopathies of different genetic causes.

**TABLE 3:** Phenotypic characteristics of inherited cardiomyopathies between human and adult zebrafish models.

Cardiomyopathy type	Hallmarks in human	Similar features in adult zebrafish models	Surrogate features in adult zebrafish models
HCM	(1) Diastolic dysfunction (2) Increased ventricular wall thickness (3) Cardiomyocyte hypercontractility (4) Fetal gene reactivation	(1) Diastolic dysfunction (2) Cardiomyocyte hypercontractility (3) Fetal gene reactivation	(1) Increased ventricular surface area (2) Increased papillary muscle density and ventricle trabeculation (3) Increased cardiomyocyte cell size
DCM	(1) Systolic dysfunction (2) Chamber dilation (3) Cardiomyocyte hypercontractility (4) Fetal gene reactivation	(1) Systolic dysfunction (2) Cardiomyocyte hypercontractility (3) Fetal gene reactivation	(1) Increased ventricular surface area (2) Reduced papillary muscle density (3) Reduced swimming capacity
RCM	(1) Increased myocardium stiffness (2) E/A ratio > 2 (3) Diastolic dysfunction	Not reported yet	Not reported yet
ACM	(1) Ventricular dysfunction and structural alterations (2) Ventricular arrhythmia (3) Fibrofatty infiltration	(1) Heart enlargement (2) Bradycardia	(1) Peripheral edema (2) Marked myocyte action potential remodeling (3) Epicardial fat tissue accumulation

*ACM, arrhythmogenic cardiomyopathy; DCM, dilated cardiomyopathy; HCM, hypertrophic cardiomyopathy; RCM, restrictive cardiomyopathy.*

### ACM Genes

To date, more than 15 genes have been linked to ACM, of which five genes encode desmosomal proteins located in the intercalated disk, including desmoplakin (*DSP*), desmoglein-2 (*DSG2*), desmocollin-2 (*DSC2*), plakophilin-2 (*PKP2*), and plakoglobin (*JUP*) (McKoy et al., 2000; Rampazzo et al., 2002; Gerull et al., 2004; Pilichou et al., 2006; Syrris et al., 2006; Vimalanathan et al., 2018; Roberts et al., 2019). Mutations in *PKP2*, *DSP*, and *DSG2* are the most common causes, accounting for about 45% of all ACM probands, and all other genes are relatively rare (Padron-Barthe et al., 2017). Thus far, *JUP* and integrin-linked kinase (*ILK*) are the two human ACM genes that have been modeled in adult zebrafish.

#### JUP

Plakoglobin encodes plakoglobin, a member of the catenin protein family. A homozygous 2-base pair deletion (2057del2) in *JUP* results in premature truncation of plakoglobin protein and was the first sequence variant linked to ACM (McKoy et al., 2000). Asimaki et al. (2014a) generated a cardiomyocyte*-*specific transgenic fish that harbored human *JUP* cDNA containing the 2057del2 mutation. The transgenic mutant fish survived to adulthood and exhibited cardiac phenotypes, including an enlarged chamber size, wall thinning, bradycardia, and increased mortality rate. There were marked abnormalities in action potentials and ionic currents in ventricular myocytes, which likely contributed to arrhythmogenesis in this model. Asimaki et al., further conducted a high-throughput screening to search for bioactive compounds that attenuated disease phenotypes. They successfully identified SB216763, an activator of the canonical Wnt/β-catenin signaling pathway, which suppresses the ACM-like phenotypes.

#### ILK

Integrin-linked kinase encodes a serine/threonine protein kinase which participates in cell matrix interactions and induction of biomechanical signals for cytoskeleton remodeling, cell survival, differentiation, and proliferation (Wu, 2001). Brodehl et al. (2019) characterized phenotypes of transgenic fish overexpression of human ILK variants p.H77Y and p.P70L identified from cohorts with arrhythmogenic cardiomyopathy. Because fewer than 20% of p.P70L and p.H77Y zebrafish could survive after 15 days, the authors mainly performed phenotypic characterization at the embryonic stage such as 3 days post-fertilization. Significant reduction of fractional shortening (FS) and mild reduction of action potential were noted. Adult histology and cardiac morphology studies showed epicardial fat tissue accumulation in the transgenic fish hearts, replicating the phenotypic trait of fibrofatty infiltration in human ACM.

### Cardiomyopathy Genes With Complex Syndromes

Because cardiomyopathy is a highly heterogeneous disease, overlapping phenotypes often coexist. For example, ventricular arrhythmias occur in up to 40% of patients diagnosed with DCM (Spezzacatene et al., 2015). Several adult zebrafish models for other inherited cardiac syndromes concurrent with cardiomyopathy are described below.

#### SCN5A

The *SCN5A* gene encodes the alpha subunit of the cardiac sodium channel, Na_V_1.5, which has a central role in controlling cardiac excitability. Variants in *SCN5A* have been linked to a spectrum of different human cardiac arrhythmia syndromes (Wilde and Amin, 2018). For example, a missense mutation, D1275N, in the *SCN5A* gene has been associated with various cardiac phenotypes, including atrial standstill and enlargement, sinus node dysfunction, tachyarrhythmias, conduction disease, and DCM (McNair et al., 2004). Huttner et al. (2013) generated a transgenic zebrafish line harboring an *SCN5A-D1275N* mutation *Tg*(*SCN5A-D1275N*) and then characterized the cardiac phenotypes by using video microscopy and electrocardiography (ECG). They found that the adult zebrafish had bradycardia, conduction system abnormalities, and premature death. Later, Yan et al. (2020) used an iWorx-based ECG system (iWorx) to study episodes of sinus arrest in *Tg*(*SCN5A-D1275N*) as an inherited model for sick sinus syndrome. Interestingly, they also noted sinus arrest episodes in a small subpopulation of wildtype fish. This subpopulation of wildtype fish manifests unique phenotypic traits of sick sinus syndrome, such as increased QRS/P ratio and chronotropic incompetence, that are different from the *Tg*(*SCN5A-D1275N*) model.

#### KCNJ8

*The KCNJ8* gene encodes the pore-forming subunit of adenosine triphosphate (ATP)–sensitive potassium channels (K_ATP_). The V65M variant in the *KCNJ8* gene was associated with Cantú syndrome, a rare genetic condition characterized by congenital hypertrichosis and cardiovascular abnormalities including an enlarged, hypercontractile heart (Brownstein et al., 2013; Cooper et al., 2014). Tessadori et al. (2018) reported a *KCNJ8 V65M* knock-in model in adult zebrafish. The heterozygous knock-in zebrafish had substantially enlarged ventricles and enhanced cardiac output and contractile function, which was similar to the characteristic signs in patients with Cantú syndrome. Through confirming the causality of the *KCNJ8 V65M* mutation, this study provided the feasibility of modeling patient-specific mutations in adult zebrafish via knock-in technology.

#### PITX2

*PITX2*, located in locus 4q25, encodes a transcription factor that is critical in establishing the left-right axis and in the asymmetrical development of internal organs and, thus, confers major susceptibility to atrial fibrillation. Collins et al. (2018, 2019) generated a loss-of-function allele of *pitx2c* using site-specific transcription activator-like effector nucleases (TALEN) in zebrafish and assessed its adult cardiac phenotypes. By using bright-field mode imaging, pulsed-wave Doppler imaging of blood flow, and ECG, the authors detected cardiac dysfunction, arrhythmias, and atrial conduction defects in homozygous *pitx2c* mutant fish and a subset of heterozygous *pitx2c* mutant fish. They further showed that changes in sarcomeric and metabolic gene expression and function preceded the onset of cardiac arrhythmia in the *pitx2c* mutant fish, suggesting that developmental perturbations predispose to functional defects in the adult heart. This is the first attempt to utilize adult zebrafish to model atrial fibrillation–like diseases of humans.

## New Inherited Cardiomyopathy Genes

More than 100 genes have been linked to cardiomyopathies, but these genes can only explain about 75% of HCM, 50% of DCM, 40% of RCM, and 60% of ACM (Groeneweg et al., 2015; Harakalova et al., 2015; Gallego-Delgado et al., 2016; Makavos et al., 2019; Rosenbaum et al., 2020). More genetic factors need to be discovered. Historically, most causative genes were identified from linkage analysis of cardiomyopathy patient cohorts. More recently, NGS has become the main technology for discovery of novel genetic loci associated with cardiomyopathy (Norton et al., 2012; Ho et al., 2015; Shah et al., 2020). As a supplement to these human genetics-based approaches, Ding et al. (2013, 2016) developed a novel mutagenesis screen-based forward genetic approach in adult zebrafish for identifying genetic factors of cardiomyopathy. *DNAJB6* and *SORBS2* were identified as new candidate cardiomyopathy susceptibility genes, which were supported by evidence from sequencing data from human patients.

### DNAJB6

The *DNAJB6* gene encodes a member of the J protein family that functions as a molecular chaperone to facilitate protein folding and protein quality control (Hageman et al., 2010; Kampinga and Craig, 2010). In mammals, there are two DNAJB6 isoforms that result from alternative splicing of the same gene. The short DNAJB6(S) isoform is encoded by cDNA that includes the first 6 exons and manifests a cytoplasmic expression pattern. In contrast, the longer DNAJB6(L) isoform is encoded by cDNA that includes an additional two exons at the 3′-end of the gene and manifests a nuclei and/or endoplasmic reticulum-specific expression pattern (Hanai and Mashima, 2003; Ding et al., 2016). Mutations in *DNAJB6*(*S*) have been previously linked to limb-girdle muscular dystrophy type 1D (LGMD1D) in humans (Sarparanta et al., 2012). Different from the short *DNAJB6*(*S*) isoform, *DNAJB6*(*L*) is a cardiac-enriched isoform that is primarily disrupted in *GBT411*. Whereas the heterozygous *GBT411* exaggerates DIC phenotypes, homozygous *GBT411* mutant exhibits cardiac chamber enlargement and cardiac muscle disarray phenotypes at 1 year of age. The latter observation prompted Ding et al to scan human cardiomyopathy patients, leading to identification of several rare variants at the C-terminus of the *DNAJB6*(*L*) gene. Pathogenicity of the p.S316W variant has been confirmed by generation of a transgenic fish line harboring the p.S316W variant, which exaggerates DIC. In summary, the *DNAJB6*(*L*) was the first cardiomyopathy gene discovered from a mutagenesis screen in adult zebrafish.

### SORBS2

*SORBS2* is an intercalated disk protein that expresses predominantly in cardiac muscle. Prompted by the modifying effects of *GBT002* on DIC, Ding et al. (2020) studied cardiac phenotypes in a *Sorbs2* knockout mouse. Whereas heterozygous *Sorbs2*^±^ exerts deleterious modifying effects on DIC, homozygous *Sorbs2*^–/–^ manifests ACM-like phenotypes, including enlarged right ventricle, arrhythmia, and fibrosis, yielding a new ACM model. Furthermore, five rare *SORBS2* variants were identified in a cohort of 59 patients with ACM, among which two splice variants were classified as likely to be pathogenic. Although more human genetic evidence from larger cohorts is needed, these data suggested *SORBS2* was a candidate gene for ACM susceptibility.

## Use of the Adult Zebrafish Model for Cardiomyopathy Research

Since the first report of cardiac remodeling in an adult zebrafish heart, (Sun et al., 2009) the adult zebrafish has emerged as an alternate vertebrate model for studying cardiomyopathies. The development of clustered regularly interspaced short palindromic repeats (CRISPR) Cas9 technology has revolutionized precise genome editing, making for ready generation of knockout, knock-in, and/or transgenic lines harboring a particular sequence variant (Shah et al., 2015; Cornet et al., 2018; Wu et al., 2018). The Table 1 shows the various current zebrafish models for inherited cardiomyopathies. Many phenotyping toolkits have been developed for the adult zebrafish heart (Table 2), which enable differentiating causative cardiomyopathy phenotypes. Below we list three research fields that may benefit from integrating the adult zebrafish model.

### Genotype-Based Therapeutics for Inherited Cardiomyopathies

To implement genotype-based precision medicine, it is desirable to systematically generate models for each of those cardiomyopathy genes and then to assess and prioritize candidate therapeutic pathways. While the induced pluripotent stem cell (iPSC) might be a more appropriate *in vitro* model for drug screening, this type of research in the *in vivo* animal systems such as rodent models can be prohibitively expensive. The efficient zebrafish model shall accelerate this research direction. Besides the existing panel of inherited cardiomyopathy models in adult zebrafish (Table 1), more models will likely be generated in the upcoming decade. Shih et al. (2015) systematically searched zebrafish homologs of 51 DCM genes and identified corresponding homologs for 49 genes, which shall contribute to this research direction.

Among well-known therapeutic pathways, some broadly target various forms of cardiomyopathy, whereas others target cardiomyopathies with a known specific cause. For example, a series of genetic studies uncovered the therapeutic effects of inhibiting the mammalian target of rapamycin (mTOR) in anemia-induced cardiomyopathy, DIC, a *bag3* knockout cardiomyopathy model, and a *lamp2* cardiomyopathy knockout model (Ding et al., 2011, 2016, 2019; Dvornikov et al., 2019). Possibly, dysregulated mTOR signaling is a common pathologic event among cardiomyopathies of different etiology, as suggested by other reports of studies in mammalian cardiomyopathy models, and mTOR inhibition is a common therapeutic strategy (McMullen et al., 2004; Marin et al., 2011; Ramos et al., 2012; Sciarretta et al., 2018). In the *desmoplakin*-based ACM model, canonical Wnt/beta-catenin signaling was reduced. Activation of Wnt signaling through the administration of a GSK3b inhibitor, SB216763, reversed the ACM disease phenotype in ACM models including the zebrafish model (Asimaki et al., 2014b; Chelko et al., 2019). The roles of Wnt/beta-catenin in other adult zebrafish models of cardiomyopathies remain to be investigated. It is expected that more research on genotype-based therapeutics in zebrafish will inform the development of precision medicine for each type of inherited cardiomyopathy.

### Assessing Sequence Variants to Find Novel Cardiomyopathy Genes

To advance genotype-based precision medicine, the remaining unknown causative cardiomyopathy genes need to be identified. With the rapid advent of NGS techniques, human genetic studies of cardiomyopathy cohorts are identifying many new candidate genes. Moreover, results from human genomic studies are identifying large numbers of sequence variants from known causative genes; however, most identified sequence variants have not been characterized, making them of variants of unknown significance (VUS). The zebrafish will likely be used increasingly as an *in vivo* model for testing candidate genes and those VUS because of its smaller size, lower cost, and higher throughput than rodent models. Because many sequence variants are missense mutations, loss-of-function knockout mutants could result in negative conclusions or provide misleading information. As shown by recent studies of sequence variants from *GATAD1, KCNJ8*, and *DNAJB6*(*L*) genes (Ding et al., 2016; Yang et al., 2016; Tessadori et al., 2018), knock-in or transgenic technology can be used to precisely model missense mutations.

### Modifier Screening for Isolating Novel Cardiomyopathy Genes and Therapeutic Targets

In addition to validating candidate genes identified in human genetic studies, genetic studies in adult zebrafish can uncover new susceptibility genes. A forward mutagenesis screening approach has been shown to be effective for identifying modifier genes for DIC (Ding et al., 2016). Interestingly, all three deleterious mutants identified from that approach, *ANO5, DNAJB6*(*L*), and *SORBS2*, were either causative or susceptibility genes for cardiomyopathies (Wahbi et al., 2013; Ding et al., 2020). Indeed, the border between modifier genes and causative genes could be blurred-it has been increasingly recognized that multiple genetic lesions might coexist in cardiomyopathy patients and contribute synergistically to the severity of pathogenesis (Ingles et al., 2005; Kelly and Semsarian, 2009; Gifford et al., 2019).

From a modifier screen, two types of modifier mutants could be identified: those that exerted deleterious modifying effects and those that exerted salutary modifying effects. Whereas the former type identifies susceptibility genes, the latter type could suggest therapeutic target genes. As shown by studies of *GBT419/rxraa*, Ma et al. (2020) established retinoid X receptor alpha a (*rxraa*) as a new therapeutic target gene for DIC and reported the underlying mechanism. Through leveraging the integrated *loxP* sites in the insertional vector, they uncovered a spatiotemporally predominant mechanism of *rxraa*-based therapy, i.e., that endothelial-specific *rxraa* activation, but not myocardial- or epicardial-*rxraa* activation, conferred therapeutic effects on DIC (Ma et al., 2020). Besides identifying therapeutic target genes via harnessing salutary modifiers, mechanistic studies of deleterious modifiers could also identify a particular way to manipulate the modifier gene to exert therapeutic effects. Ding et al. (2016) conducted detailed studies of *GBT411/dnajb6b*(*L*) and found that while *dnajb6b*(*L*) loss-of-function exerts deleterious effects, overexpression of *dnajb6b*(*L*) in cardiomyocytes exerts therapeutic effects on DIC. The therapeutic effects of *dnajb6b*(*L*) overexpression were confirmed in a mouse DIC model by using an adeno-associated virus (AAV) 9–based gene delivery system.

## Limitations

Although adult zebrafish can be a model for cardiomyopathy studies, this lower vertebrate model is not without limitations. Despite a much more economic model than mouse, an adult zebrafish reaches sexual maturity in 3 months, which is actually longer than mouse. Different from the 4-chambered heart structure in humans, a zebrafish heart consists of two chambers. Its large atrium has a more active role during the cardiac cycle than atria of mammals. On Doppler echocardiography, the ratio between the E-wave and the A-wave in an adult zebrafish is less than one, suggestive of higher active filling (A-wave) than passive filling (E-wave) (Lee et al., 2014). A zebrafish heart does not have a clearly defined ventricular wall. Instead, it consists of highly trabeculated ventricular myocardium surrounded by a small compact layer of cardiomyocytes (Hu et al., 2000). Therefore, differences in hemodynamic properties of zebrafish vs. human myocardium must be considered in using this model. Throughout their lifecycle, zebrafish maintain considerable capacity for generating cardiomyocytes, whereas mammals lose this capability within a few days of birth (Poss et al., 2002; Doppler et al., 2017). This contribution of cardiomyocyte proliferation to cardiac remodeling needs to be considered (Sun et al., 2009).

Although about 70% of human genes have at least one obvious zebrafish ortholog, the remaining 30% of human genes do not have an obvious zebrafish ortholog, making it impossible to study these genes in zebrafish (Howe et al., 2013). Zebrafish have also undergone an additional whole-genome duplication (i.e., teleost-specific genome duplication); therefore, about 15% of human genes are associated with more than one zebrafish gene (average, 2.3) (Meyer and Schartl, 1999; Howe et al., 2013). However, cardiomyopathy genes might have a higher degree of conservation, as indicated by human DCM genes, 96% of which have a zebrafish ortholog (Shih et al., 2015). Shih et al. (2015) were able to prioritize one of many homologs for most DCM genes for further genetic manipulation based on their relative expression in the embryonic heart, adult heart, and adult somites.

## Conclusion

**FIGURE 1:**
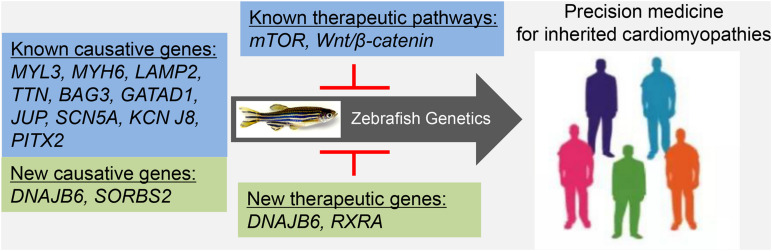
Future perspective. Adult zebrafish can be used to generate genotype-specific cardiomyopathy models, to identify genotype-specific therapeutic pathways, to discover new causative genes, and to discover new therapeutic genes. Discoveries from this efficient vertebrate model might inform the development of precision medicine for inherited cardiomyopathies.

Despite these limitations, in the last decade, adult zebrafish have emerged as an important vertebrate model for studying inherited cardiomyopathies. The use of zebrafish is not limited to modeling known genotype-based cardiomyopathies for therapeutic development and has already been extended to discovering new susceptibility genes and therapeutic target genes (Figure 1). In the future, this small tropical fish holds the promise of contributing substantially to implementing genotype-based precision medicine for inherited cardiomyopathies.

## Author Contributions

YD and XX designed the review outline and wrote the review. YD and HB did the literature search, data extraction, and interpretation. All authors contributed and approved the final version of the manuscript.

## Conflict of Interest

The authors declare that the research was conducted in the absence of any commercial or financial relationships that could be construed as a potential conflict of interest.
